# Early positivity signals changes in an abstract linguistic pattern

**DOI:** 10.1371/journal.pone.0180727

**Published:** 2017-07-05

**Authors:** Júlia Monte-Ordoño, Juan M. Toro

**Affiliations:** 1 Universitat Pompeu Fabra, Barcelona, Spain; 2 ICREA, Pg. Lluís Companys, Barcelona, Spain; Waseda University, JAPAN

## Abstract

The extraction of abstract structures from speech (or from gestures in the case of sign languages) has been claimed to be a fundamental mechanism for language acquisition. In the present study we registered the neural responses that are triggered when a violation of an abstract, token-independent rule is detected. We registered ERPs while presenting participants with trisyllabic CVCVCV nonsense words in an oddball paradigm. Standard stimuli followed an ABB rule (where A and B are different syllables). Importantly, to distinguish neural responses triggered by changes in surface information from responses triggered by changes in the underlying abstract structure, we used two types of deviant stimuli. Phoneme deviants differed from standards only in their phonemes. Rule deviants differed from standards in both their phonemes and their composing rule. We observed a significant positivity as early as 300 ms after the presentation of deviant stimuli that violated the abstract rule (Rule deviants). The amplitude of this neural response was correlated with participants’ performance in a behavioral rule learning test. Differences in electrophysiological responses observed between learners and non-learners suggest that individual differences in an abstract rule learning task might be related to how listeners select relevant sources of information.

## Introduction

Language proficiency involves the ability to understand and create novel sentences not encountered or produced before. Putatively, such ability depends on the acquisition of abstract rules according to which a language is organized [1]. For example, at the sentence level, affirmative sentences in English are produced following the SVO (Subject-Verb-Object) word order. Once this order has been established, its variables can take any acceptable value and produce a valid English sentence. Similarly, at the word level, affixation rules allow for the creation of words by adding morphemes to word stems (e.g. adding the affix “-ed” to a regular verb will create a past tense of it). Because combinations of stems and affixes (just as combinations of subjects, verbs and objects) can use any acceptable values to create valid words and sentences; they are considered as examples of a token-independent pattern. Processing of such abstract patterns in speech seems to recruit dedicated neural circuits (e.g. [2]). However, even though much interest has been raised by the mechanisms responsible of learning abstract structures, there is still much to be learned from how our brain discovers the relevant regularities in the signal. For instance, although it has been suggested that both infants and adults extract this kind of information incidentally [3,4] it is still an open question whether the brain can detect violations in such abstract patterns pre-attentively or whether additional attentional or prosodic cues are needed. More importantly, it would be interesting to identify neural markers that could predict successful performance in the processing of abstract linguistic regularities. In the present study we contribute to these issues by characterizing the neural responses linked to the violation of target abstract structures. We thus will explore the neural responses that are triggered when a violation of an abstract pattern is detected and contrast them with the responses triggered when a violation of surface phonetic regularities are detected.

Marcus, Vijayan, Rao and Vishton [3] designed a study to explore if pre-verbal infants were already able to detect token-independent rules in speech. The authors created nonsense words in which syllables followed a given abstract rule (for example, ABB, where the second and third syllables were the same, generating nonsense words such as *wofefe*), and seven-month-old infants were familiarized with them during two minutes. After familiarization, novel words that either followed the same ABB rule or that followed an inconsistent rule (e.g. ABA) were presented. Infants’ looking times increased after inconsistent words, suggesting they were able to detect the abstract differences between novel consistent and inconsistent words. Studies using near-infrared spectroscopy have shown that even neonates are able to respond to such abstract rules. Different neural activations have been observed for sequences of words following a repetitive abstract pattern from sequences of words without such a pattern [5]. This suggests that the brain mechanism that is responsible for the detection of abstract regularities is already present from a very early age. Even more, there is empirical evidence that similar brain regions (more specifically, the left inferior frontal region) are engaged in the processing of artificial grammars and natural languages (see [6]). Providing evidence that the study of artificial language could help to study the processes engaged in natural language.

Previous studies have analyzed the electrophysiological markers of abstract rule learning during artificial language processing. However, different experimental methodologies and different abstract structures targeted have yielded several distinct ERP components. Tabullo and collaborators [7] created an artificial grammar to explore the role of expectancy in the modulation of neural responses. The authors observed a P300 and a N400 component in response to grammar violations followed by a later positivity. The functional properties of the late positivity were studied in comparison to the syntactic P600 that has been observed during language processing. Similar late positivities were observed in a subsequent study exploring both artificial and natural language [8]. The authors interpreted them as instances of P600 elicited by expectancy violations (for similar results in the visual domain see [9]). Even more, Bahlmann, Gunter and Friederici [10] also observed a late positivity when they presented participants with errors in a Finite-state grammar (FSG) and in a Phrase structure grammar (PSG). Following a different strategy, De Diego-Balaguer, Toro, Rodriguez-Fornells and Bachoud-Lévi [11] familiarized participants with a stream of nonsense words defined by long-distance dependencies. The authors observed an N400 component that was related to learning the nonsense words composing the artificial language. More closely linked to the present study, in an experiment by Sun, Hoshi-Shiba, Abla and Okanoya [12] participants first learned an abstract rule (similar to the rules used in [3]) from nonsense words and then were presented with new tokens following the same or a different rule. The experiment consisted of 12 blocks of a learning and a test phase with a final behavioral test. Probably because participants in their study were attending the stimuli and should pass a test phase several times, the authors did not observe the posterior component related to attentional mechanisms that had been observed in previous studies. Instead, the authors found a negative response around 400 ms that was associated with learning the rules taught to the participants.

In the present study we take a complementary approach at the study of the neural responses involved during rule learning. We will use ABB and ABA rules similar from the ones used in Marcus et al. [3] and in Sun et al. [12]. However, here we will focus on whether the brain can quickly detect surface and structural changes in an abstract rule, and how it reacts to such unexpected changes. For this, we will take advantage of the oddball paradigm. This consists on the presentation of sequences of highly frequent (standard) stimuli occasionally interrupted by infrequent (deviant) stimuli. Because deviant stimuli differ in one or more features from standard stimuli, they might trigger ERP components related to change detection (e.g. MMN and P300). The mismatch negativity (MMN) signals the rapid (pre-attentional) detection of changes in a sequence of sounds (for a review see [13]). Some studies have reported a MMN component triggered by frequency and intensity changes in tones [14] or by changes on the direction of frequency modulated sweeps [15]. In a linguistic context, the MMN has been observed after grammatical agreement violations [16]. More closely linked to the present study, it has also been observed after changes in abstract regularities in artificial languages [17]. On the other hand, the P300 has been related to a reorientation of attention towards an unexpected event [18]. This component has been observed when structural changes are introduced in a sequence of sounds [19] and after the presentation of grammatically illegal words [7]. There is thus evidence linking the P300 to the detection of abstract rules in linguistic stimuli. In the auditory domain, the Oddball paradigm is typically used to study the detection of changes in physical features such as frequency or intensity of sounds. However, it has recently been used to study the detection of more abstract, second order, features of the stimuli (e.g. [14, 15, 17]). Thus the oddball paradigm can be used to study the discovery of abstract rules in speech and the electrophysiological changes generated when a deviation is detected. To distinguish neural responses triggered by changes in phonemes from responses triggered by changes in abstract structure, in the present study we will use two types of deviant stimuli. One type of deviant stimuli (Phoneme deviants) differs from standard words only in their phonemes, while the other type of deviant stimuli (Rule deviants) differs in both their phonemes and their abstract structure. Our prediction is that only Rule deviants might trigger responses after the detection of changes in an abstract pattern. We also ran a behavioral rule learning test to explore possible correlations between the neural responses observed and the overt generalization responses produced by the participants.

## Methods

### Participants

Thirty-seven undergraduate students (20 female) from the Center for Brain and Cognition database participated in the experiment (mean age = 20.57, SD = 2.11). All of them were right-handed, balanced bilingual Catalan-Spanish speakers (they were exposed to both languages from an early age and received a bilingual formal education). None of the participants reported any auditory problem. Participants signed a written informed consent form and were paid for their participation in the experiment. Two participants were excluded from the analysis due to problems related to the ERP recording. Two participants did not complete the behavioral test due to technical problems.

### Stimuli

One hundred and twenty standard stimuli were created. They consisted on trisyllabic CVCVCV (consonant-vowel) nonsense-words. Syllables used to create words were *fa*, *fe*, *fo*, *ma*, *me*, *mo*, *pa*, *pe*, *po*, *ra*, *re*, *ro*, *ta*, *te*, *to*. These syllables were combined following an ABB pattern, so the second syllable was repeated (creating words like *feroro*, *morara*, *tapepe*; see Table 1). Deviant stimuli were created using a different set of new syllables: *ke*, *ki*, *ku*, *le*, *li*, *lu*, *se*, *si*, *su*. There were two types of deviant stimuli, Phoneme deviants and Rule deviants. Phoneme deviants were 36 new nonsense words that followed the same ABB pattern as the standard stimuli (e.g. *kilulu*, *lesisi*, *likeke*; see Table 1). Rule deviants were 36 new nonsense words that followed a novel ABA pattern (the first syllable was the same as the third one; e.g. *kiluki*, *lesile*, *likeli*; see Table 1). Thus, all deviant stimuli were made by different syllables from standard stimuli, but only Rule deviants followed a different abstract pattern. All words were synthesized using MBROLA [20] with an Italian female database (it4). Each phoneme had its fundamental frequency set to 240Hz and duration of 120 msec. The MBROLA speech synthesizer has the advantage of allowing for a very tight control of the duration and pitch (fundamental frequency) of each phoneme used. Since we set the duration of each phoneme to 120 ms, we were able to time the onset of the third syllable in each word with precision.

**Table 1:** Stimuli used during ERP recording.

Standards		Phoneme deviants		Rule Deviants	
*rofefe*	*tomeme*	*lesisi*	*sikeke*	*lesile*	*sikesi*
*rofafa*	*terara*	*lesusu*	*sikuku*	*lesule*	*sikusi*
*romeme*	*teroro*	*lekiki*	*sulele*	*lekile*	*sulesu*
*ropepe*	*tefafa*	*lekuku*	*sulili*	*lekule*	*sulisu*
*retoto*	*tefofo*	*lisese*	*sukiki*	*liseli*	*sukisu*
*retata*	*taroro*	*lisusu*	*sukeke*	*lisuli*	*sukesu*
*refafa*	*tarere*	*likeke*	*kelili*	*likeli*	*kelike*
*remomo*	*tafefe*	*likuku*	*kelulu*	*likuli*	*keluke*
*ratoto*	*tafofo*	*lusese*	*kesisi*	*luselu*	*kesike*
*rafefe*	*tameme*	*lusisi*	*kesusu*	*lusilu*	*kesuke*
*rafofo*	*tamomo*	*lukiki*	*kilele*	*lukilu*	*kileki*
*rameme*	*fotete*	*lukeke*	*kilulu*	*lukelu*	*kiluki*
*ramomo*	*fotata*	*selili*	*kisese*	*selise*	*kiseki*
*rapepe*	*forere*	*selulu*	*kisusu*	*seluse*	*kisuki*
*rapopo*	*forara*	*sekiki*	*kulele*	*sekise*	*kuleku*
*torere*	*faroro*	*sekuku*	*kulili*	*sekuse*	*kuliku*
*torara*	*farere*	*silele*	*kusese*	*silesi*	*kuseku*
*tofafa*	*fatoto*	*silulu*	*kusisi*	*silusi*	*kusiku*

*Note*. The table presents Phoneme deviants, Rule deviants and 36 (out of 120) standard words used during the experiment. Deviant stimuli were constructed using novel phonemes. Both standards and Phoneme deviants followed an ABB rule. Rule deviants followed an ABA rule. Notice that the table only shows a selection of 36 of the standard stimuli. All the syllable combinations were used to create the 120 total standards used in the experiment.

### ERP recording

The EEG was recorded using an elastic cap of 32 channels (actiCAP) using the Modified Combinatorial Nomenclature (MNC) system. As a result, 28 electrodes were recorded from the scalp (Fp1, 2; F3, 4, 7, 8; Fz, FC1, 2, 5, 6; T7, 8; C3, 4; Cz; CP1, 2, 5, 6; TP9, 10; P3, 4, 7, 8; Pz; Oz). Two more electrodes were placed to the left and right mastoid (M1 and M2) and to control the ocular movements and blinking, two different electrodes were placed on the outer side (HEOG) and below (VEOG) the right eye. In addition, an electrode placed on the tip of the nose was used as an online reference. The signals were sampled at a rate of 500 Hz. The electrode impedances were kept under 10kΩ and EEG was recorded during the familiarization phase of the experiment.

### Procedure

Throughout the experiment participants sat comfortably in a soundproof room and heard the stimuli through two loudspeakers. Participants watched a silent movie and were asked not to pay attention to the auditory stimuli. Similar designs have been used in previous studies registering ERP and passive rule learning tasks (e.g. [14]). Also, learning of abstract rules as the ones implemented in our experiment has been observed independently of focused attention (e.g. [4]). The presentation of the stimuli was structured in 2 blocks with a break of about 2 minutes between them. During each block 552 words were presented. Of these, 480 were standard words (all the standard stimuli repeated four times) and 72 were deviant stimuli (36 phoneme deviants and 36 rule deviants, each presented only once). The overall deviant probability was set to 0.13. Thus, although both blocks contained deviant stimuli, the first block was expected to serve as an acquisition phase, while learning should be consolidated during the second block. We thus expected to find more reliable effects during the second block as participants should have extracted and processed the abstract rule after the first block (see also [11, 12]). Order of presentation of the standard stimuli was randomized, only avoiding immediate repetitions of each item. The order of presentation of the two types of deviants (phoneme and rule) was randomized. Deviant stimuli were presented after a minimum of five standard stimuli. Stimulus onset asynchrony (ISO) was set to 1000 ms. Once the presentation of stimuli was over, participants performed a behavioral test to assess the rule learning of the participants (for a similar design see [11–12]). During the test, 8 random pairs of deviant stimuli were presented. In each pair there was a phoneme deviant and a rule deviant word (ISO was set to 1000 ms). Each phoneme deviant was paired with its equivalent rule deviant (e.g. *kisese* was paired with *kiseki*; see Table 2). Participants were asked to choose which word was more similar to the stimuli presented during the familiarization. The experimental procedure was approved by the ethical committees of the funding body (European Research Council) and the Universitat Pompeu Fabra.

**Table 2:** Stimuli used in the behavioral test.

Correct	Incorrect
*kisese*	*kiseki*
*sikeke*	*sikesi*
*lekuku*	*lekule*
*lisese*	*liseli*
*lesisi*	*lesile*
*lekiki*	*lekile*
*kulili*	*kuliku*
*kilele*	*kileki*

*Note*. Two types of test stimuli were presented. The correct stimuli followed the same ABB rule as standards. The incorrect stimuli followed an ABA rule. Each time a new random selection of deviant stimuli was used.

### ERP analysis

ERP data were offline processed using Brain Vision Analyzer (v2, Brain Products) software. The data was offline band-pass filtered from 1 to 30 Hz (12 dB) and re-referenced to the average of the linked mastoids. Epochs of 550 ms were used with a baseline from -100 to 0ms relative to the third syllable onset. Ocular correction was applied using “ICA ocular correction” function of Brain Analyzer 2, where an extended Informax algorithm is used, and epochs with an amplitude >10 0μV at EOG channels and with an amplitude >100μV at EEG channels were rejected. The criterion we used to include a participant in the analysis was to have a minimum of 80% of valid trials. The mean percentage of trials kept per subject was of 94.98% for the standard stimuli, of 98.6% for the Phoneme deviant stimuli, and of 98.34% for the Rule deviant stimuli. The analysis was carried out using a cluster mass permutation test [21] run in Matlab (The Mathworks) software. In this procedure the non-averaged ERP data is analyzed and the mean difference between standards and deviants is calculated for every channel-data point pair. These pairs are clustered on the basis of temporal and spatial adjacency and their cluster-level statistic is calculated. Then, a random partition is repeated several times for every cluster and the cluster-level p-value is obtained. One thousand permutations were run to assess the probability of finding clusters with higher statistics if the standard and deviant stimuli were assigned randomly. Because we had different types of deviant stimuli we performed 2 comparisons: Standard vs Phoneme Deviants and Standard vs Rule Deviants. We selected four regions of interest (ROIs) for the analysis: fronto-central left (electrodes F3, FC1, FC5, C3), fronto-central right (electrodes F4, FC2, FC6, C4), centro-parietal left (electrodes CP1, CP5, P7, P3), centro-parietal right (electrodes CP2, CP6, P4, P8). That is, we run a Permutation analysis in each ROI separately and then each channel-data point pair was analyzed in the electrodes included in the ROI without averaging their data. Sun et al. [12] found that the ERP effects were more salient in later blocks during their experiment. That is the reason why we examined the data corresponding to the second block in order to ensure that we were working with the optimal performance of the participants.

## Results

### Behavioral results

The results from the rule learning test revealed that the participants correctly learned the rule implemented over standard words. Participants correctly identified (M = 60.22, SD = 23.89; t(32) = 2.45, p = .020; performance expressed as the percentage of correct responses) test items abiding to the rule over test items following a different pattern.

### ERP results

**Fig 1:**
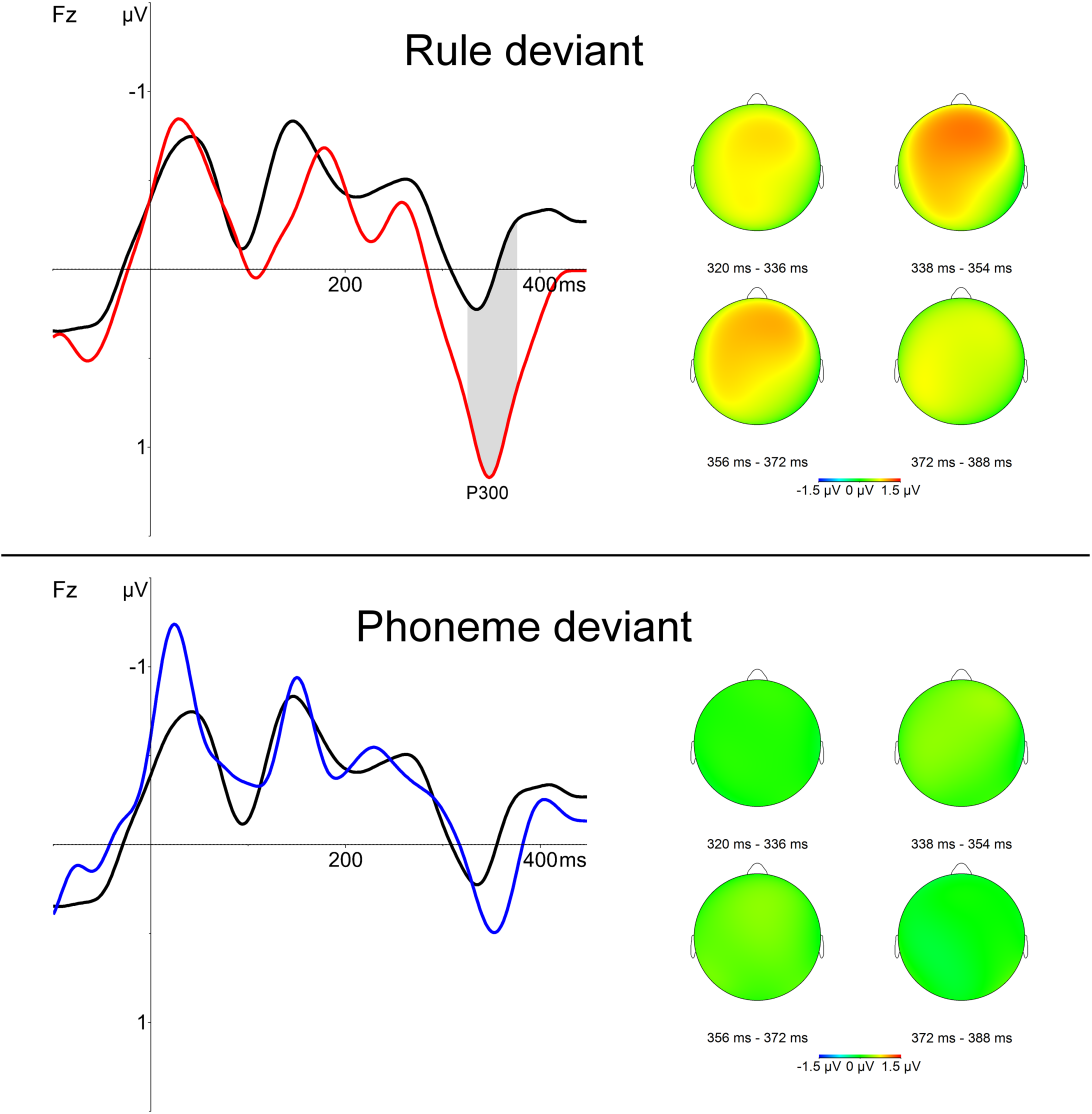
Grand average ERPs and polarity maps for rule and phoneme deviants. ERP graphs reflect the neural responses registered in Fz electrode after standard stimuli (black line), rule deviants (red line) and phoneme deviants (blue line). Polarity maps reflect the activity for all the participants during the P300 time window. The upper panel corresponds to the presentation of deviant stimuli differing in both phonemes and rule from standard stimuli (rule deviants). The lower panel corresponds to the presentation of deviant stimuli differing only in phonemes from standard stimuli (phoneme deviants). A positivity is observed after 300 ms in left frontal region after the presentation of rule deviants.

**Fig 2:**
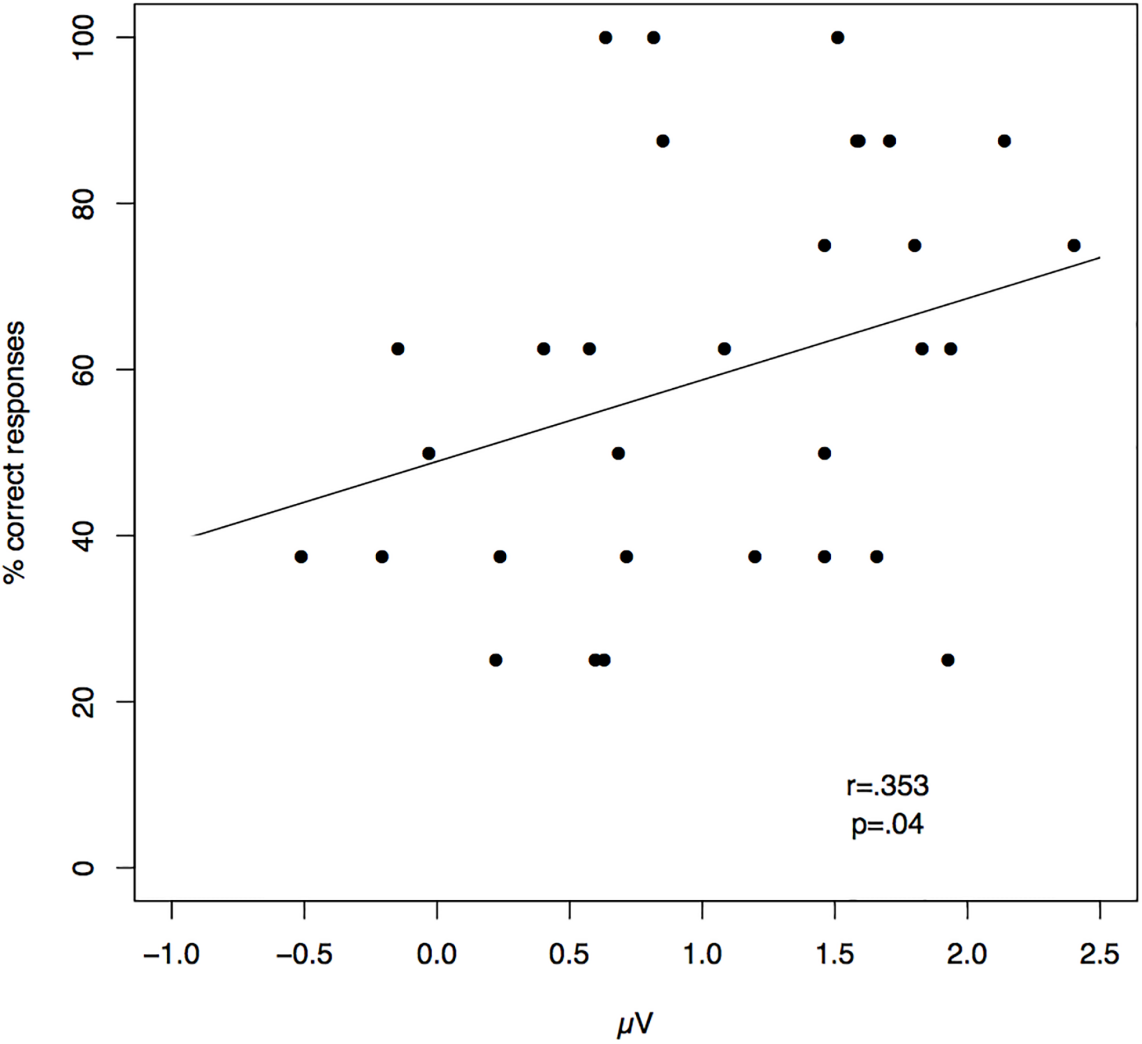
Correlation between the mean amplitude of the P300 component and the performance in the behavioral test. The mean amplitude of the P300 component at the target ROI (Left Fronto-Central) during the rule deviant presentation positively correlated with the participants’ performance in the rule learning test. This correlation was performed with the amplitude difference between the component after rule deviant stimuli and after standards.

The comparison between standards and Phoneme deviants did not yield any significant difference between them. That is, the presentation of novel words abiding to the rule did not trigger any distinct neural response when compared with the presentation of standard words. In contrast, the comparison between standards and Rule deviants showed a significant positivity from 328 to 378 ms in the fronto-central left region (cluster mass permutation test p < .05; see Fig 1). No other significant differences were observed. Previous studies have reported a correlation between the amplitude of ERP components and behavioral performance in tasks assessing the detection of abstract regularities in artificial languages (e.g. [11]). We thus conducted a correlation analysis for the amplitude of the P300 we observed and performance in the behavioral rule learning test. Interestingly, we found a positive correlation between the amplitude of the positivity observed after the presentation of Rule deviants and performance in the test (*r* = .353, *p* = .04; see Fig 2 and table C in S1 File). Participants who performed better in the generalization test showed a more salient P300 response when deviant stimuli varying in abstract structure were presented.

### Learners vs. non-learners

To further explore the correlation between neural responses and rule learning (as measured in the behavioral test), we divided the group of 33 participants into a group of learners (participants who obtained a minimum of 60% in the behavioral test, N = 19; M = 77.63, SD = 14.17) and a group of non-learners (N = 14; M = 36.60, SD = 9.12). In the group of learners we observed a significant positivity from 330 to 386 ms (cluster mass permutation test, p < .01) and a marginally significant negativity from 416 to 450 ms (cluster mass permutation test, p = .071) in the left fronto-central region after Rule deviants. No significant differences were observed after Phoneme deviants (Please see Figure A in S1 File). The permutation analysis performed over the group of non-learners did not show any significant differences between standards and Rule deviants. There was, however, a marginally significant negative response from 384 to 408 ms in the left centro-parietal region (cluster mass permutation test, p = .065) after Phoneme deviant stimuli (please see Figure B in S1 File).

Next, we explored the possible differences between learners and non-learners during the P300 time window (from 320 to 380 ms). We conducted a repeated measures ANOVA with the within-subject factors Region (Fronto-central, Centro-parietal), Hemisphere (Left, Right), Block (1, 2) and Stimulus (Phoneme deviant, Rule deviant), and the between-subjects factor Group (Learner, Non-learner). We used peak voltages extracted from the difference waves resulting from the activations after standards and the two types of deviants. The electrodes included in the analyses were the same used in the ROIs of the Permutation analysis: Fronto-central left ROI (F3, FC1, FC5, C3), Fronto-central right ROI (F4, FC2, FC6, C4), Centro-parietal left ROI (CP1, CP5, P7, P3) Centro-parietal right ROI (CP2, CP6, P4, P8). Then the data from each channel was averaged for each ROI (for a similar procedure see [22]). The p values were corrected using the Greenhouse-Geisser adjustment. The Bonferroni correction was applied on multiple comparisons. The results showed a significant main effect of block (F(1,31) = 4.77, p < .05). We also observed a Region x Stimulus interaction (F(1,31) = 6.84, p < .05), and the pairwise analysis showed that after the Rule deviants there was a greater P3 effect size in the Fronto-central region in comparison to the Centro-parietal region, although this result was marginally significant (p = .051). Moreover, we observed a significant Block x Region x Hemisphere interaction (F(1,31) = 4.67, p < .05). The pairwise comparisons showed that in the Centro-Parietal region, in the left hemisphere the effect from Block 2 was greater than during the Block 1 (p = .044). More interesting, a significant Block x Hemisphere x Stimulus interaction was also observed (F(1,31) = 4.269, p < .05). The pairwise comparisons indicated that in the left hemisphere, the P3 effect size after the rule deviant presentation was greater during block 2 than during block 1 (p = .006). Even more, we observed a significant Block x Stimulus x Group interaction (F(1,31) = 4.24, p < .05), showing that the P3 component observed after the Rule deviants was significantly greater in the group of learners than in the group of non-learners during the second block (p = .01).

## Discussion

The main goal of the present study was to explore the neural events that are triggered by the detection of an abstract rule violation. We observed a positive response around 330 ms elicited after the presentation of deviant words that followed a different abstract pattern from standards (Rule deviants), but not after deviant words that followed the same patterns (Phoneme deviants). Even more, the amplitude of the positivity was correlated with the behavioral performance of the participants in the rule learning test.

Timing of the positive response we observed after Rule deviants is congruent with the P300 component. The frontal distribution of the positivity we observed differs from the centro-parietal distribution of the classic P300, or at least from the P3b subcomponent. However, the P3a subcomponent, frontally distributed, match with our results. The P3a has been proposed to reflect the detection of novelty in a sequence of stimuli [23] and it has been observed even when changes take place in task-irrelevant sounds [18]. In the present study we observed a positivity only after the presentation of deviant stimuli that differed from standards in both the phonemes used to create the words and the abstract structure they followed (Rule deviant stimuli). That is, the positivity was linked to a violation of the abstract rule, not only to a change in phonemes. Because the P3a component is suggested to signal the reorientation of attention [18, 23], its emergence in the present task could reflect that the detection of an abstract rule violation engaged the participants' attention. We also observed that the amplitude of this positivity was correlated with behavioral performance in a rule learning test. The better a participant performed in the behavioral rule learning test, the more amplitude in the positivity was registered.

In an artificial grammar learning task, Tabullo and collaborators [7] observed two positivities after grammatically illegal words; the first positivity was interpreted as a P3b component and was observed in learners and non-learners group, while the second positivity was only present in the learners group and was interpreted as a member of the P600 component (for similar results, see [8]). The authors interpreted that this response was triggered by unexpected events (sound sequences with low probability of occurrence), and signaled the detection of a rule violation. Moreover, the authors observed that while the early positivity was observed in both learner and non-learner groups, the late positivity was significant only in the learner group. This later positivity was also observed in other studies also using artificial language [9, 24] or artificial grammar tasks [10]. However, we did not observe a P600 component. This could be due to differences in the complexity of the pattern the participants had to discover from the input. In the current study, we presented abstract rules based on adjacent repetitions (e.g. ABB) that could be applied to any combination of syllables and that are supposedly detected by efficient processes [25]. In this way, our stimuli mirrored those created by Marcus et al. [3] and Sun et al [12]. Previous studies reporting a P600 have focused on non-adjacent AxB rules [7] or even central embedded rules (e.g. [10]) that are putatively more demanding. Thus, although it has been suggested that P600 and P300 reflect similar processes related to the detection of violations in structured stimuli, the P600 could reflect the processing of more complex structures [9]. Such differences in complexity could lead to the observation of earlier responses (around 300 ms) after the violations of abstract repetitive rules as the ones used here.

Deviant stimuli often trigger a mismatch negativity (MMN) response when presented in an oddball paradigm (e.g. [14–15, 17]). However, in the present study we did not observe a MMN after neither Phoneme nor Rule deviants. Lack of this component is consistent with previous studies exploring early markers of abstract rule violations (e.g. [11, 19]). For instance, Tabullo and collaborators [7] did not observe a MMN component even though they had behavioral data indicating that participants had learned the rule defining the words in their artificial grammar. The authors suggested that the lack of a MMN component was explained because, despite the fact that participants had learned the grammar-like rules, they were not proficient in them. Pointing in the same direction, Friederici, Steinhauer and Pfeifer [24] showed that only L2 learners who had a highly proficient level in their second language showed a MMN component after being presented with syntactic violations in their L2. It is thus an open question whether different experimental procedures, likely involving heavy training with the target rules composing the nonsense words, would yield a MMN component after the presentation of deviant items. Although it is worth mentioning that this study did not use an Oddball Paradigm, and this could also have a role in the results observed, another possibility is that violations of abstract rules as the ones implemented in the present study do not trigger an early mismatch response. Bekinschtein et al. [19] proposed that the MMN and the P300 might involve the detection of auditory changes at different levels; a local and a global level. A local change would consist on a modification of the physical characteristics of the auditory stimulus. Changes at this token-specific level would trigger a MMN. In contrast, a global change would consist on a modification in the abstract regularities defining a sequence of events. Changes at this token-independent level would trigger a P300 response (see also [26]). So, even after heavy training with target abstract rules, test items implementing a different rule might not trigger a MMN response. Further experiments using training will help to disentangle these different interpretations.

In the current study, and that of Bekinschtein and collaborators [19], only the participants who had acquired the rule showed a P300 component. Moreover, we observed that this component correlated with the performance in the behavioral rule learning test. The group of non-learners, in contrast, showed a marginally significant negativity after the presentation of the deviant stimuli that share the structure with standard stimuli (Phoneme deviants). This component was triggered around 400 ms in the posterior electrodes. This neural response has been associated with lexical processing (e.g. [27, 28]) and lexical search [29]. The negativity around 400 ms might suggest that participants in our study who did not learn the rule were likely engaging in lexical recognition processes instead of abstract pattern generalization. In fact, De Diego-Balaguer and collaborators [11] also observed an N400 component that was triggered in a word learning task over nonsense words similar to the ones used here. Importantly, during the present experiment, participants were not given any instruction regarding the kind of information they should extract from the words. In fact they were asked to focus their attention in the silent film. We decided to play a silent video during the experiment because learning of this type of abstract rules has been observed independently of focused attention (e.g. [4]). Thus, in the case of non-learners, they might have prioritized lexical over structural cues. This strategy might have prevented them from learning the abstract (token-independent) rule. In contrast, participants who learned the rule seem to have engaged in abstract generalization processes (as signaled by the positivity observed after 300 ms). Moreover, the learner group also showed a frontally distributed marginally significant N400 effect after the rule deviant stimuli. The frontal N400 component has been observed during the processing of structural linguistic information [30–31]. In fact, in the experiments by Sun and collaborators [12] and Tabullo and collaborators [7] a frontally distributed N400 was observed after the presentation of rule or grammar violations. This provides further evidence that learners and non-learners in our experiment might be applying different learning strategies over the stimuli presented; one group might have focused on the lexical information and the other might have focused on the abstract structures (see also [32–33] for evidence regarding how the distribution of the N400 might vary depending on the learning task performed).

The possibility that learners and non-learners were focusing on different sources of information during the present task poses several questions for further research. Studies have demonstrated individual differences during the extraction of information from speech that are linked to more general cognitive abilities. The detection of non-adjacent regularities that are important to learn (among others) morphological rules has been found to correlate with auditory discrimination abilities [17], and individual differences in second language learning are related to the extent to which a listener is able to efficiently process sounds in her native language [34]. It would thus be interesting to explore the extent to which individual differences in the discovery of abstract structures in speech might be linked to basic auditory processes. It might be the case that a better categorization of the elements composing a sequence results in more efficient abstraction and generalization (for results pointing in this direction see [35–37]). Even more, individual differences in rule learning might result from listeners focusing on different aspects of the information present in the speech signal. Recent experiments have shown that learners are specially tuned to discover abstract regularities among nonsense stimuli only when the stimuli are interpreted in a communicative context. When the same stimuli are processed in a non-communicative context, it is difficult to extract abstract rules over them ([38]; see also [39]). Similarly, listeners find it more difficult to learn abstract rules similar to the ones used here over consonants than over vowels [40], likely because the listeners are focusing on the consonants’ lexical role [41]. Individual differences in learning abstract rules might thus be in part related to the extent to which the learner can take advantage of the appropriate sources of information present in the signal. Focusing on abstract, token-independent structures instead of concrete, token-specific features of the stimuli might have led a group of participants in the present study to learn the rules (as reflected by both results in the behavioral test and the emergence of the P3 component). In contrast, results suggest that the group of non-learners might have focused on surface features (the phonemes) that differentiated individual nonsense words. Differences across groups suggest that focusing on different cues present in the signal (phonetic versus structural) might be at the base of learning differences that are marked by specific neural responses.

## Conclusion

In the current study, we explored the rapid detection of rule violations in an Oddball paradigm. We used two kinds of deviant stimuli, one changing only in the phonemes (Phoneme deviant) and the other changing also in the rule (rule deviants). We observed that a P300 component was elicited only after Rule deviant stimuli and was correlated with the behavioral performance of the participants in the rule learning test. This is in line with some studies showing that P300 is related with the processing of structure information. Moreover, two marginal components were observed only in the particular study of the groups of learners and non-learners. The learner’s group elicited a frontal N400 component after the presentation of rule deviants, while the non-learner’s group elicited a parietal N400 component after the presentation of phoneme deviants. Differences in electrophysiological responses observed between learners and non-learners suggest that individual differences in an abstract rule learning task might be related to how listeners select relevant sources of information from the signal. Our results thus demonstrate early responses triggered by changes in an abstract rule and identify processes that might underlie individual differences in the detection of abstract structures.

## Supporting information

S1 File Supplementary note. We have included as supporting information additional figures showing separately the results for the group of learners and non-learners (Figure A and B). We have also included supplementary tables with the peak values of the difference waves for the P300 effect during block 1 (Table A) and block 2 (Table B), and the results from the behavioral test with their corresponding amplitudes in the target ROI during the P300 time window (Table C).
(DOCX)
