# Supplementary material for: Early positivity signals changes in an abstract linguistic pattern
Source: PLoS One. 2017 Jul 5;12(7):e0180727. doi: 10.1371/journal.pone.0180727 (PMC5498064; doi:10.1371/journal.pone.0180727)
Supplement: S1 File — We have included as supporting information additional figures showing separately the results for the group of learners and non-learners (Figure A and B). We have also included supplementary tables with the peak values of the difference waves for the P300 effect during block 1 (Table A) and block 2 (Table B), and the results from the behavioral test with their corresponding amplitudes in the target ROI during the P300 time window (Table C). (DOCX) [file pone.0180727.s001.docx]

*Supporting Information*

Early positivity signals changes in an abstract linguistic pattern

Júlia Monte-Ordoño & Juan M. Toro

Correspondence to: juanmanuel.toro@upf.edu

**Figure A. Grand average ERPs and polarity maps of learner group**


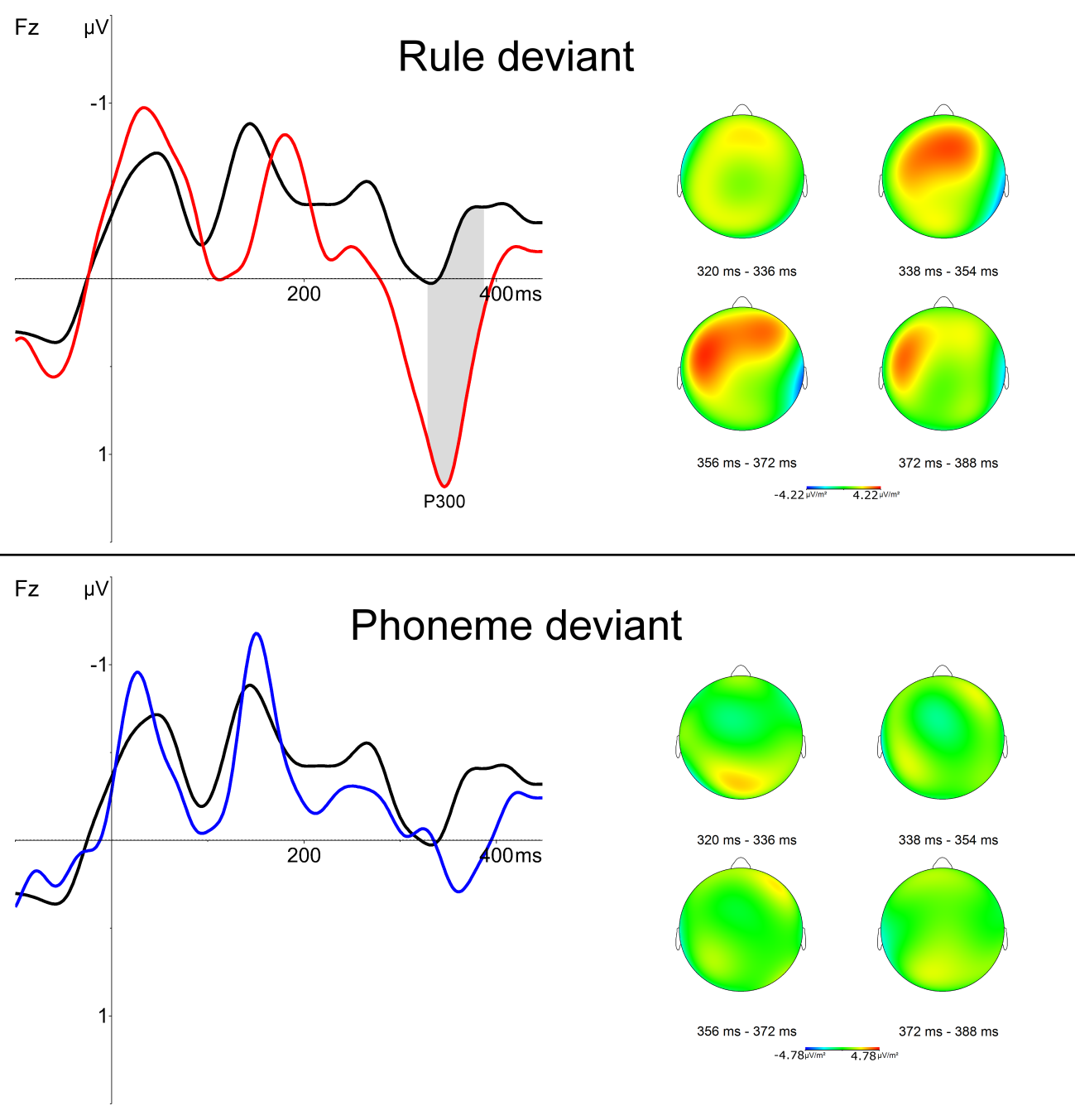


ERP graphs reflect the neural responses registered in Fz electrode after standard stimuli (black line), rule deviants (red line) and phoneme deviants (blue line) for the learner group. Polarity maps reflect the activity for learner group during the P300 time window. A positivity is observed after 300 ms in left frontal region after the presentation of rule deviants.

**Figure B. Grand average ERPs and polarity maps of non-learner group**


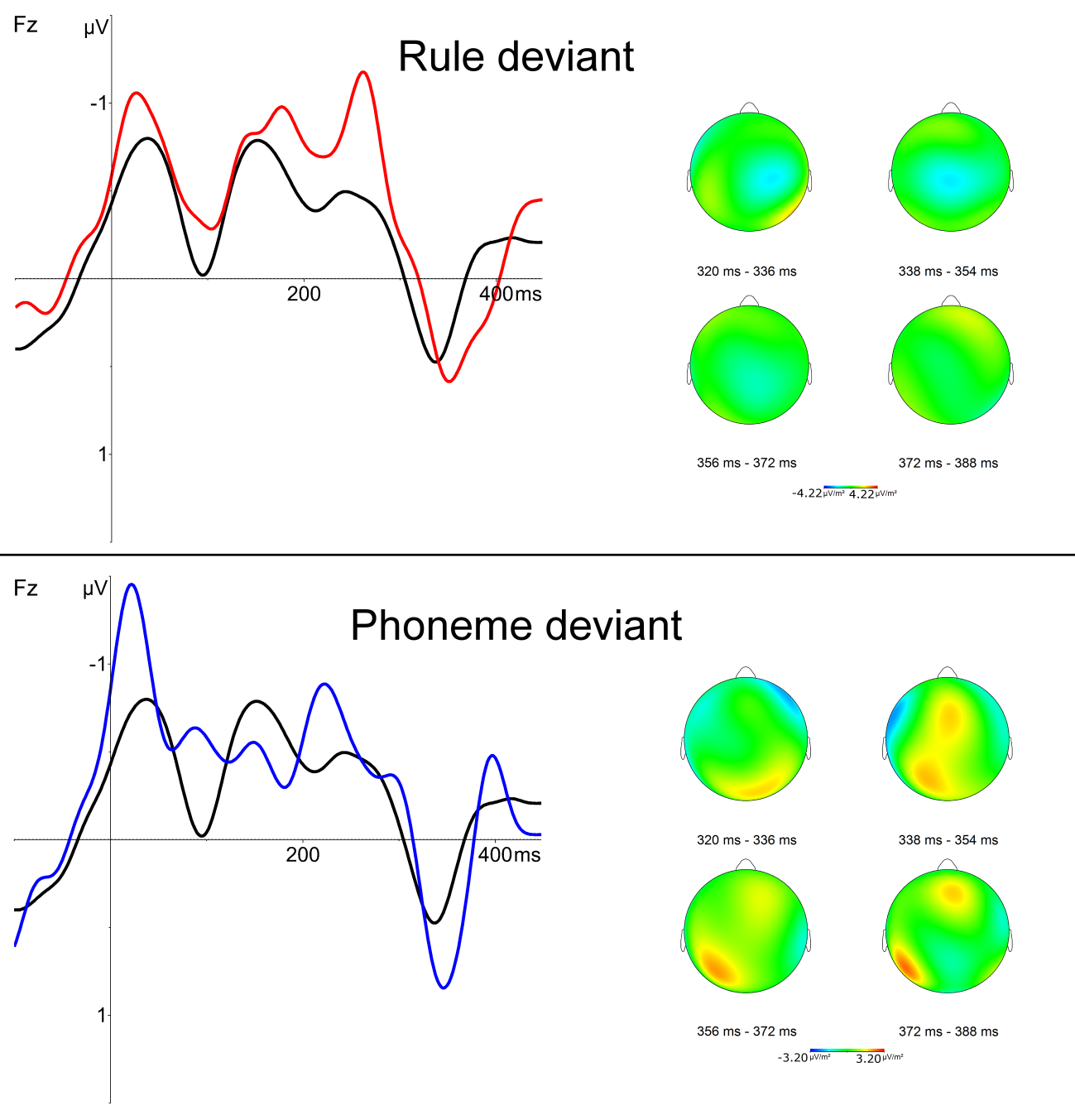


ERP graphs reflect the neural responses registered in Fz electrode after standard stimuli (black line), rule deviants (red line) and phoneme deviants (blue line) for the non-learner group. Polarity maps reflect the activity for non-learner group during the P300 time window. No positivity is observed after 300 ms in left frontal region after the presentation of rule deviants.

**Table A. Peaks of the difference waves of the ROIs during block 1**

|  | Fronto-central | | | | Centro-parietal | | | |
| --- | --- | --- | --- | --- | --- | --- | --- | --- |
|  | Left hemisphere | | Right hemisphere | | Left hemisphere | | Right hemisphere | |
| Group | Phoneme | Rule | Phoneme | Rule | Phoneme | Rule | Phoneme | Rule |
| 1 | 2.1376 | 1.5198 | 2.0489 | 1.2391 | 1.6104 | .554 | 1.339 | .6159 |
| 1 | 1.7707 | 1.5556 | 2.6391 | 1.6847 | 1.4411 | 1.7139 | 1.2108 | 1.646 |
| 0 | 1.319 | .5076 | .4246 | .7968 | 2.3157 | .5067 | 1.6829 | .5057 |
| 0 | 2.0367 | 2.038 | 1.2855 | 1.7089 | 1.0659 | .9801 | 1.0618 | .5166 |
| 0 | .6507 | 1.7259 | .7396 | 1.958 | 1.2326 | .6441 | .5574 | 1.7253 |
| 0 | .9605 | 1.7952 | .5995 | 1.7219 | 1.8557 | .6345 | 1.737 | 1.0143 |
| 1 | 2.1514 | 1.4812 | 2.0436 | .1225 | 2.1571 | 3.6569 | 1.8091 | 2.3054 |
| 0 | .8847 | 1.1938 | 1.1627 | .8612 | .9475 | 2.1206 | 1.2732 | .7259 |
| 0 | .695 | 1.1148 | .9847 | .711 | .907 | 1.1213 | 1.2096 | .5827 |
| 1 | .8475 | 1.8153 | .6437 | 1.9372 | .9705 | 2.047 | 1.4396 | 2.0655 |
| 0 | .4749 | .9408 | .8266 | .6175 | .2705 | 1.5379 | .9136 | .9423 |
| 1 | 1.7761 | 2.0616 | 1.2025 | 2.1131 | .6743 | 2.1404 | 1.4194 | 2.0654 |
| 1 | .956 | 1.3856 | 1.4603 | 1.9736 | 1.0326 | 1.5822 | 1.4185 | 1.3189 |
| 0 | .2284 | 1.6242 | .1726 | .6195 | .5274 | 1.7599 | .4532 | -.2618 |
| 0 | -.4477 | 1.5449 | .4752 | .6839 | -.6305 | 1.3412 | .8248 | .0399 |
| 1 | 2.0762 | 1.2495 | 1.8582 | 1.5344 | 1.8057 | .8013 | 3.2367 | 1.1154 |
| 1 | .3389 | 1.9699 | 1.0452 | 2.6337 | -.1246 | .9164 | 1.5251 | 1.9115 |
| 1 | 1.5191 | 1.1621 | 1.5825 | .7585 | .2915 | .7303 | .2959 | .9656 |
| 1 | 1.8608 | 1.22 | 2.969 | 1.4099 | .8918 | 1.6923 | 1.6594 | 1.0768 |
| 0 | 1.0953 | 1.3138 | 1.3001 | .7371 | 1.6761 | 2.1817 | 2.1181 | 1.4949 |
| 0 | 1.8433 | 1.609 | 1.5818 | 1.6676 | 1.6656 | 1.5023 | 1.0423 | 1.1288 |
| 1 | .4516 | .6081 | .788 | .6353 | 1.3379 | .6862 | 1.3672 | 1.4294 |
| 1 | 1.4367 | 1.3568 | 1.7422 | .7037 | 2.7886 | -.0082 | 2.7469 | .1477 |
| 0 | 1.3835 | .4994 | 1.6624 | 1.112 | 1.2698 | .7588 | 2.5514 | 2.3469 |
| 1 | 2.2661 | 1.9995 | 2.4469 | 2.1161 | 1.7625 | 1.0478 | 1.3071 | .8329 |
| 0 | .7729 | .4393 | 2.3552 | 1.444 | .93 | .3805 | 2.4402 | .9052 |
| 1 | .5545 | .8246 | 1.4364 | .716 | 1.657 | 1.3392 | 1.0537 | 1.0581 |
| 1 | 1.8433 | 1.609 | 1.5818 | 1.6676 | 1.6656 | 1.5023 | 1.0423 | 1.1288 |
| 0 | 1.8433 | 1.609 | 1.5818 | 1.6676 | 1.6656 | 1.5023 | 1.0423 | 1.1288 |
| 1 | 1.6433 | 1.7046 | 1.7991 | 2.4412 | 1.449 | .7159 | 2.1197 | 2.0154 |
| 1 | -.0798 | 1.0878 | .8857 | .8247 | .2962 | 1.2739 | 1.1465 | .6624 |
| 1 | .2365 | .6989 | 1.2912 | .6055 | 1.0218 | .9183 | 1.0285 | .7602 |
| 1 | 1.2599 | 1.843 | 1.7566 | .928 | .8825 | 3.4592 | 2.3751 | 2.3638 |

Group 1 corresponds to learner and group 0 corresponds to Non-learner.

**Table B. Peaks of the difference waves of the ROIs during block 2**

|  | Fronto-central | | | | Centro-parietal | | | |
| --- | --- | --- | --- | --- | --- | --- | --- | --- |
|  | Left hemisphere | | Right hemisphere | | Left hemisphere | | Right hemisphere | |
| Group | Phoneme | Rule | Phoneme | Rule | Phoneme | Rule | Phoneme | Rule |
| 1 | .847 | 1.8285 | 1.3969 | 1.777 | 2.0505 | 2.3293 | 1.9152 | 2.4002 |
| 1 | .9523 | .8516 | 1.3872 | .359 | 1.5213 | .5992 | .9738 | -.2743 |
| 0 | .2288 | .5965 | .9965 | .496 | .0529 | .1758 | .6966 | .1405 |
| 0 | .4275 | -.0305 | -.005 | -.0373 | .7993 | .2241 | .4597 | .7336 |
| 0 | .6558 | 1.6589 | .0794 | .8704 | 1.4631 | .8571 | .459 | 1.3746 |
| 0 | -.0974 | .6309 | -.2628 | .656 | .3769 | .7143 | .9902 | 1.0661 |
| 1 | .6027 | 1.0844 | .5556 | 1.2525 | .467 | .6653 | -.0187 | 1.2494 |
| 0 | .0495 | -.2073 | -.1122 | .3367 | -.1657 | -.1882 | .7946 | -.1717 |
| 0 | 2.0531 | .7153 | 1.1838 | .0359 | 2.6013 | .5486 | 1.5704 | -.1063 |
| 1 | -.8325 | .4023 | -1.1674 | .409 | -.6788 | .2976 | -.4914 | -.2196 |
| 0 | 1.1975 | .685 | 1.2063 | .9 | 1.4999 | .3329 | 1.5807 | .7726 |
| 1 | 1.1045 | 2.4027 | .6145 | 2.534 | .9225 | 2.1455 | .945 | 1.2912 |
| 1 | 1.1211 | -.1472 | 1.7606 | 2.5234 | 1.0729 | -.4064 | 2.896 | .7009 |
| 0 | 2.5213 | 1.9272 | 2.6717 | 2.0186 | 1.9494 | 1.717 | 1.9979 | 1.5791 |
| 0 | 2.1695 | -.5115 | .1329 | -.2936 | 2.0428 | .0864 | .4228 | .1643 |
| 1 | 2.4588 | 1.8008 | 1.5764 | .2123 | 1.3968 | -.1017 | 1.2374 | .904 |
| 1 | 1.7055 | 1.9372 | 1.3936 | 1.4094 | .9942 | 1.8103 | 1.607 | 2.6954 |
| 1 | 2.1356 | 1.5821 | 2.1924 | 1.0693 | 2.3775 | 1.4081 | 1.5597 | 1.7413 |
| 1 | 1.1427 | .5744 | 1.2761 | .8795 | .7278 | .3479 | -.3951 | 1.0081 |
| 0 | 1.1404 | .2376 | 1.3505 | 1.3088 | -.1511 | .738 | .5975 | 1.5528 |
| 0 | 1.1752 | 1.4612 | 1.3174 | 1.3142 | .9274 | .484 | 2.338 | -.2984 |
| 1 | .1057 | .6364 | .6241 | 1.4469 | 1.3963 | .6552 | .9531 | 1.4514 |
| 1 | .9941 | 3.0035 | 1.3393 | 2.8361 | 1.1729 | 1.5937 | 1.577 | 1.4638 |
| 0 | 1.3258 | 1.1988 | 1.2372 | 1.3605 | .9887 | .4583 | 2.6369 | 1.4843 |
| 1 | .8521 | 2.7227 | 2.0572 | 4.5212 | .6352 | 1.1907 | 1.4111 | .8668 |
| 0 | .9723 | .2225 | .5365 | .6132 | 1.947 | .4757 | 1.6811 | 1.1444 |
| 1 | 2.3747 | 2.1402 | .927 | .3985 | 4.1296 | .314 | 1.7444 | 1.3137 |
| 1 | 1.1752 | 1.4612 | 1.3174 | 1.3142 | .9274 | .484 | 2.338 | -.2984 |
| 0 | 1.1752 | 1.4612 | 1.3174 | 1.3142 | .9274 | .484 | 2.338 | -.2984 |
| 1 | 1.2793 | 1.511 | 1.1541 | 1.896 | .8839 | 1.7247 | 1.2208 | 2.6829 |
| 1 | .8272 | 1.5907 | .7627 | 1.8449 | .5174 | .3848 | 1.6036 | .781 |
| 1 | .2902 | .8175 | 1.2632 | .3587 | .0998 | .5567 | .5251 | .1022 |
| 1 | -.1678 | 1.7064 | -.3974 | 1.7085 | .4413 | 1.7833 | .0641 | 2.3376 |

Group 1 corresponds to learner and group 0 corresponds to Non-learner.

**Table C. Behavioral test results and the corresponding amplitudes in the Fronto-central left ROI during the P300 time window**

| Responses (%) | Fronto-central Left (μV) |
| --- | --- |
| 62.5 | 1.8285 |
| 87.5 | .8516 |
| 25 | .5965 |
| 50 | -.0305 |
| 37.5 | 1.6589 |
| 25 | .6309 |
| 62.5 | 1.0844 |
| 37.5 | -.2073 |
| 37.5 | .7153 |
| 62.5 | .4023 |
| 50 | .685 |
| 75 | 2.4027 |
| 62.5 | -.1472 |
| 25 | 1.9272 |
| 37.5 | -.5115 |
| 75 | 1.8008 |
| 62.5 | 1.9372 |
| 87.5 | 1.5821 |
| 62.5 | .5744 |
| 37.5 | .2376 |
| 50 | 1.4612 |
| 100 | .6364 |
| 62.5 | 3.0035 |
| 37.5 | 1.1988 |
| 75 | 2.7227 |
| 25 | .2225 |
| 87.5 | 2.1402 |
| 37.5 | 1.4612 |
| 75 | 1.4612 |
| 100 | 1.511 |
| 87.5 | 1.5907 |
| 100 | .8175 |
| 87.5 | 1.7064 |
